# Diet-Related Mobile Apps to Promote Healthy Eating and Proper Nutrition: A Content Analysis and Quality Assessment

**DOI:** 10.3390/ijerph18073496

**Published:** 2021-03-28

**Authors:** Jihye Choi, Chongwook Chung, Hyekyung Woo

**Affiliations:** 1 Department of Health Promotion and Behavioral Sciences, University of Texas Health Science Center at Houston School of Public Health, Houston, TX 77030, USA; jennchoi1222@gmail.com; 2 Department of Biomedical Engineering, Ulsan National Institute of Science and Technology, Ulsan 44919, Korea; cogpsydoc@gmail.com; 3 Department of Health Administration, Kongju National University, Chungnam 32588, Korea

**Keywords:** content analysis, diet, nutrition, MARS, mobile apps, quality assessment

## Abstract

Dietary mobile applications (apps) continue to hold promise for facilitating a healthy diet and managing nutrition. However, few studies have objectively evaluated the content and quality of such apps in Korea. The present study assessed the content and quality of dietary mobile apps using the Mobile App Rating Scale (MARS). We selected 29 dietary apps based on keywords and eligibility criteria for inclusion in the analyses. We conducted regression analyses to examine the association between app content and MARS scores. Most of the apps featured a tracking tool, while few featured rewards or follow-up management. Our quality assessment revealed that the top-rated apps have distinct levels of quality in terms of MARS scores. The regression analyses showed that the ways in which the apps provide information and motivate the users are statistically significant predictors of app quality. Our findings may facilitate the selection of dietary apps in Korea and provide guidelines for app developers regarding potential improvements in terms of content and quality.

## 1. Introduction

Increased awareness and the high burden of comorbidities associated with an unhealthy diet have highlighted the importance of healthy eating [1]. Indeed, healthy eating is central to the prevention and management of chronic diseases and is a critical element in longevity. Although the importance placed on specific nutrients varies by population [2], diet and dietary patterns are directly linked to health outcomes, as illuminated by the proverb “we are what we eat” [3]. Many previous studies have reported that adherence to a high-quality diet or a prudent dietary pattern is inversely associated with a reduced risk of overall mortality [2,4,5]. In Korea, nutritional management was added to the 2020 National Health Plan to bolster healthy dietary lifestyles and limit the spread of chronic diseases [6].

Mobile health interventions via smartphone applications (apps) are no longer in their nascent stage; they are thriving expeditiously as promising and convenient strategies for health promotion. They allow the collection of dynamic health information, enable self-monitoring, and deliver real-time feedback outside of a clinical setting [7]. Among the many types of mobile apps available, those that help with healthy eating and weight management are most sought-after because it is relatively easy to improve these behaviors via self-monitoring, which plays a critical role in helping individuals adhere to strict dietary regimes [8,9]. Consequentially, apps focused on diet and nutrition represent the fastest-growing area of health promotion apps, as seen through their proliferation in the Apple App Store and the Google Play Store [10].

Despite consistent demand that has sustained a robust mobile communication and subsequent mobile health (mHealth) industry comprising a plethora of diet- and nutrition-related apps, researchers have only recently begun to conduct content analyses and quality assessments of these devices. Furthermore, most studies have been limited to evaluations of the frequency and intentions regarding app usage, assessments of apps as methods for delivering nutritional education material, and examinations of their feasibility [11,12,13,14,15,16]. Early users relied solely on subjective consumer ratings (e.g., star rating systems) and annotated reviews (with questionable credibility) when deciding which app to use. Consequently, users were limited in terms of their ability to select apps with features, content, and quality that were tailored to their specific needs.

In this study, we analyzed the content and quality of mobile apps focused on diet and nutrition in Korea, with the goal of assisting users in app selection. We also developed evidence-based guidelines for app developers to improve the content and quality of new and existing diet- and nutrition-related apps.

## 2. Materials and Methods

### 2.1. Design

We conducted exploratory content analyses with a sample of 29 diet- and nutrition-related mobile apps selected using relevant keywords and eligibility criteria. Following descriptive analyses of the app content, we conducted a quality assessment of the apps using the previously validated Mobile App Rating Scale (MARS) developed by Stoyanov and colleagues (2015). Finally, we performed regression analyses to examine the association between the different categories of app content and MARS scores. The unit of analysis was the individual mobile app.

### 2.2. App Selection

We used keywords to search for diet- and nutrition-related mobile apps that were commercially available on the iOS and Android platforms. The keywords *calorie* and *diet* were chosen as terms most frequently associated with *eating habits* on Google and Naver (a Korean online platform with its own search engine).

Between 26 December and 30 December 2019, we conducted an app search on the Google Play Store and the Apple App Store using four different mobile devices (Samsung Galaxy S4, Samsung Galaxy Note 8, Apple iPhone Xs Max, and Apple iPhone 8). Of the apps returned in the search, we downloaded only those apps that met all six of the following criteria, which were intended to identify the most frequently used dietary apps among users in Korea. Then these were included in our descriptive analyses of app content.

C1. Apps with ratings of four stars or higher

C2. Apps within the top 100 most reviewed apps

C3. Apps set in the Korean language

C4. Apps related to diet

C5. Apps without duplicates

C6. Apps free of charge

### 2.3. Descriptive Content Analyses

Previously, we conducted a pilot study in which we used the same keywords to identify and assess several of the most frequently downloaded diet- and nutrition-related apps. This enabled us to determine the different categories into which the app content could be classified. In the main study, we classified the app content into the following categories: behavior change techniques (BCTs), provision of information, and function [17,18]. Then the selected apps were compared across the different categories.

### 2.4. Quality Assessment of Dietary Mobile Apps

MARS is one of the most widely used multidimensional tools for evaluating the quality and content of mobile health applications [19,20]. It consists of 23 items grouped into the following five dimensions: engagement, functionality, aesthetics, information, and subjective quality. All items are scored on a 5-point scale (1: inadequate, 2: poor, 3: acceptable, 4: good, and 5: excellent), and a final mean score is given for each dimension. MARS has been previously validated in an original study [19], which indicated good reliability for the subscales (Cronbach’s alpha = 0.80–0.89) and overall scale (Cronbach’s alpha = 0.90), as well as good objectivity for the subscales (Intraclass Correlation Coefficient, ICC = 0.50–0.80) and overall scale (ICC = 0.90). In the present study, we assessed the selected apps using the 21 items from MARS between 31 December 2019 and 6 January 2021. Four blinded reviewers (rather than one single reviewer) conducted the assessment to prevent potential bias in the evaluation process. Because we were focused only on the app content, we excluded the two items that assess user opinions and/or satisfaction in a target population. We calculated the mean scores of the apps with standard deviations for each of the dimensions and conducted regression analyses to assess the association between app content and MARS scores.

## 3. Results

### 3.1. App Selection

**Figure 1:**
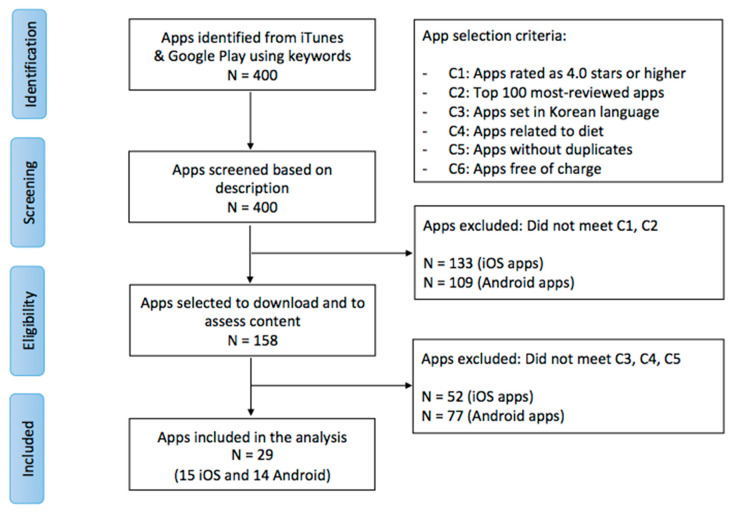
Flowchart of application (app) selection.

We identified a total of 400 mobile apps from the initial keyword search on iTunes and the Google Play Store. We applied the inclusion and exclusion criteria to screen the identified apps over two rounds. First, an app was excluded if it had a rating of less than 4.0 stars and if it was not in the top 100 apps according to the number of reviews it had received. This step led to the exclusion of 133 iOS apps and 109 Android apps, leaving a total of 158 apps to be downloaded for potential assessment of content. In the second round of screening, an app was excluded if it was not offered in the Korean language; was irrelevant to diet or healthy eating; had duplicate content, descriptions, or images from another app; or required payment to download. Following the exclusion of apps that did not meet the above criteria, 29 apps (15 iOS apps and 14 Android apps) were included in the quantitative and qualitative analyses (Figure 1).

### 3.2. Content Analyses of the Apps

Table 1 shows the selected dietary apps with content classified according to the BCT, provision of information, or function. While most of the apps primarily focused on tracking or logging features, some offered rewards or follow-up services such as post-consumption or post-activity management. App content that was classified as a BCT was further divided into two clusters, tracking and motivation, to specifically account for apps that provided tracking or logging services for nutrient intake, caloric intake, step counts, activity, food, water intake, and weight and apps that provided motivation to users through rewards, monitoring, instruction, community support, and performance management.

Of the apps for which the main content was focused on BCTs, the most were in the tracking category. This included apps used to log weight (*n* = 24, 82.8%) and caloric intake (*n* = 23, 79.3%) and to record step counts (*n* = 12, 41.4%). In the motivation category, apps that provided self-monitoring for diet and health management made up the largest group (*n* = 14, 48.3%) compared to apps that provided notifications (*n* = 2, 6.9%). In the information category, apps that provided information regarding recommended caloric intake formed the largest group (*n* = 22, 75.9%), followed by those related to nutrient intake (*n* = 13, 44.8%) and general health (*n* = 7, 24.1%). Few apps provided tailored menus or diet plans to users free of charge (*n* = 2, 6.9%). In the function category, the largest group included apps that provided diet feedback and nutritional reports based on the user’s average monthly consumption of foods and supplements (including paid apps; *n* = 20, 69%). The next highest in number were apps that provided meal reminders (*n* = 16, 55.2%), all of which were offered for free. Although all of the apps that were compatible with wearable devices were free of charge, a few had distinct functions of their own (*n* = 5, 17.2%). Table 1 summarizes the number and proportion of free and paid apps in the different content categories.

### 3.3. Quality Assessment of App Content

#### 3.3.1. MARS Score Dimensions

**Figure 2:**
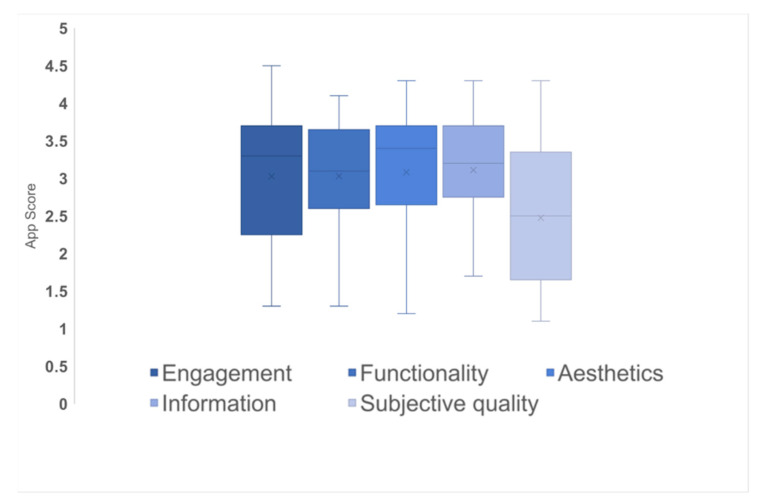
App scores according to Mobile App Rating Scale (MARS) dimensions.

Figure 2 shows the app scores according to the MARS dimensions. The mean score for all of the apps was 2.95 out of 5 (SD = 0.79). The scores for engagement ranged from 1.3 to 4.5, with a mean score of 3.0 (SD = 0.93); those for functionality ranged from 1.3 to 4.1, with a mean score of 3.0 (SD = 0.77); those for aesthetics ranged from 1.2 to 4.3, with a mean score of 3.1 (SD 0.85); those for information ranged from 1.7 to 4.3, with a mean score of 3.1 (SD = 0.73); and those for subjective quality ranged from 1.1 to 4.3, with a mean score of 2.5 (SD = 0.99). On average, aesthetics and functionality were high-scoring dimensions compared to the dimension of subjective quality. Although we selected apps based on their high star ratings and high number of reviews, app quality, as appraised by reviewers, differed significantly from that reflected in the MARS scores. 

#### 3.3.2. MARS Scores for Individual Apps: Best and Worst Apps

After calculating the overall mean quality scores for the selected apps by dimension, we examined the individual apps and determined the five best and worst apps based on their MARS scores. The five best were comparable across the dimensions, and their MARS scores corresponded with the general preferences of users. The highest-scoring app, with an overall score of 4.1 out of 5, was an Android app called God of Weight Loss—a Diary for Diet and Exercise, which scored 4.3 for engagement, 4 for functionality, 4.3 for aesthetics, 4 for information, and 4 for subjective quality. While users preferred Friends on a Diet, an iOS app, over God of Weight Loss, the former received the lowest ranking among the five best apps, with a mean score of 3.8 out of 5 across the dimensions, and a score of 4.5 for engagement, 3.6 for functionality, 3.5 for aesthetics, 3.8 for information, and 3.8 for subjective quality.

The five worst apps were those with the lowest MARS scores. The app with the lowest scores was an Android app called Freeze Weight. Interestingly, this app was the most preferred by users as it had the highest star rating and the highest number of reviews. Contrary to its popularity, this app scored 1.3 for engagement, 1.4 for functionality, 1.2 for aesthetics, 1.8 for information, and 1.2 for subjective quality, resulting in a low mean score of 1.4 across the dimensions. Individual scores for the best and worst apps by dimension are shown in Table 2.

#### 3.3.3. Association between App Content and Quality

We conducted regression analyses to examine the extent to which the app content categories were associated with the overall app quality reflected by the MARS scores. The information provision and motivation categories were statistically significant predictors (*β* = 0.573, *p* < 0.01 and *β* = 0.261, *p* < 0.05, respectively) of app quality, and the model explained 66.8% of the variance observed in the MARS scores. In contrast, the tracking category of BCTs and the function category were not statistically significant predictors of the MARS scores (*β* = 0.072, *p* = 700 and *β* = 0.090, *p* = 0.549, respectively). The results of the regression analyses are presented in Table 3.

## 4. Discussion

### 4.1. Principal Findings

As previous studies have emphasized that diet- and nutrition-related apps are useful for promoting dietary behavioral change and as effective as conventional in-person clinical care [21,22,23], we sought to examine in depth prominent features of these apps and their overall quality. Accordingly, in the present study, we aimed to generate guidelines to aid users in selecting diet- and nutrition-related mobile apps and to assist app developers in the development and rapid deployment of updates to existing apps in Korea. We conducted content analyses and quality assessment of 29 dietary mobile apps selected from the iOS and Android platforms using relevant keywords and a set of eligibility criteria.

The selected apps elicited behavioral change via tracking and motivation, such as self-monitoring, provided information pertaining to diet and nutrition, and offered various functions in support of healthy eating, such as nutrition reports. This result partly aligns with previous studies in which the most frequently identified types of behavioral techniques were goal setting, self-monitoring, and the provision of feedback [24,25], although goal setting was not an observed BCT in the selected apps in this study. When we measured app quality using MARS, the selected apps were of moderate-to-high quality across the dimensions of engagement, aesthetics, functionality, information, and subjective quality. This may be due to the initial selection of apps based on high star ratings and popularity among users. Furthermore, the motivation and information categories of the app content were significantly associated with the MARS scores. The inclusion of motivational and informative features within an app may enhance the perceived functionality, aesthetics, and engagement of the app, thereby leading to repeated use among users [26]. This finding is consistent with previous research in which apps focused on improving user motivation, self-efficacy, knowledge, and goal setting were associated with higher app quality [26,27].

There are notable differences between English-only apps and Korean apps. Dietary tracking and education were the most common features reported in previous assessments of diet and nutrition apps [28]. However, these previous studies only examined English apps, and thus excluded culturally tailored diets and nutrition-related apps designed for non-English-speaking populations with poor health literacy [28,29]. While tracking and logging were the most predominant features in the Korean apps we examined, monitoring and the provision of information about caloric intake were much more prevalent than education. Moreover, where reminders and feedback were the least frequently identified features across health and wellness apps in prior studies [30,31], meal time reminders (55.2%) and feedback in the form of monthly reports on food consumption and weight changes (69%) were leading features in the function category of the app content in the present study.

Star ratings and user reviews are valuable to both developers and potential new users because they offer a crowd-sourced indicator of the effectiveness and popularity of apps [28]. However, this indicator may not be as accurate when measuring quality. Although we selected the 29 highest-rated apps (with scores ranging from 4.0 to 5 stars) based on their popularity, number of times downloaded, and presumably equal high quality, we found distinct levels of quality in terms of MARS scores. The average MARS score of the five best and worst apps was 3.98 and 1.72, respectively, suggesting a significant gap in quality among the selected highly rated apps. For instance, although it was one of the highest-rated apps, Freeze Weight received the lowest overall MARS score. It also had a particularly low score for aesthetics, which reinforces the need for app developers to consider the role of visual attributes in attracting users [32], as well as in signaling the quality and function of apps [33]. In addition, despite the importance of the motivational content designed to facilitate healthy eating, many of the highly rated apps in the present study lacked such features. Thus, skepticism is warranted regarding the validity and reliability of star ratings.

Few dietary apps have been subjected to rigorous evaluations of content and quality [28]. In the present study, some of the highest-rated apps received low MARS scores, reflecting poor quality. This implies that star ratings, along with the number of reviews per app, may not always reflect the quality or presence of useful content and therefore may not be a reliable indicator of efficacious diet- and nutrition-related apps. App developers could address this by incorporating quality assessment criteria in their work and collaborating with host platforms to improve the rating systems. This could increase the plausibility of user ratings in terms of the relationship between app quality and content, and thus better inform new users who are seeking diet and nutrition apps that suit their specific needs.

Based on the findings of this study, we offer the following suggestions as guidelines for app users and app developers in their selection and development of diet- and nutrition-related apps, respectively. First, app users should have an increased awareness that star ratings and the concomitant anecdotal reviews may not be accurate indicators to measure quality of the apps. Because needs and perceptions of apps differ greatly between individuals, we recommend that users know their needs and consult the content description of the apps prior to their decision making rather than relying solely on star ratings and user reviews. Second, as evidenced by the statistical results, we recommend that app users consider informative and motivational features priority criteria in their decision-making process as apps with these features will likely be of high quality. Third, we suggest that app developers work in close partnership with the platforms of the Google Play Store and iTunes toward a systematic and reliable rating system, thereby reducing the apparent gap between star ratings and objective quality of the apps. Lastly, we suggest that app developers take a collaborative approach with nutritionists and dieticians and draw upon features valued by these health experts to remedy any shortcomings of the current apps. Dietary counseling advice provided by nutritionists and dietitians should not be supplanted by diet- and nutrition-related apps but rather bolster the development of the apps and propose future directions to app developers.

### 4.2. Limitations

The present study had several limitations. Effective health promotion interventions are often based on health behavior theories [34]. However, the apps evaluated in the present study lacked the use of theoretical constructs such as self-efficacy or goal setting. Although previous studies have indicated that many apps lack utility in terms of evidence-based theories regarding health behavior changes [27,35,36,37], our results would likely have been different if we had specifically searched for theory-based apps as an eligibility criterion for analyses. Another weakness of our study was that we did not consider the quality and content of apps that were rated as less than 4.0 stars. Further studies are necessary to address this. Finally, because the unit of analysis was individual apps, the study did not target users from a specific population. Future studies that take into account sociodemographic factors, health needs, and experience will provide additional insight regarding how apps differ in their perceived quality and practicality when accessed by users with different characteristics and histories.

## 5. Conclusions

It is imperative that the role of nutritionists and dietitians not be offset by diet- and nutrition-related apps; they should effectively craft a comprehensive plan to help individuals reach optimal health, working in conjunction with these experts. Accordingly, extensive research and rigorous assessments of these apps are needed to justify their adequacy. The paucity of advanced research evaluating the value of these apps in Korea calls for further scrutiny, particularly in terms of quality and content. Our study is the first content analysis and quality assessment of diet- and nutrition-related apps in Korea from both the iOS and Android platforms. We hope that our findings will facilitate users in the selection of appropriate apps for improving dietary behavior and assist app developers in the creation and modification of diet- and nutrition-related apps. Finally, we expect that our results will be helpful to mHealth researchers as they conduct similar prospective studies of health intervention apps.

## Figures and Tables

**Table 1:** Content analyses of the 29 dietary mobile apps.

Content			Android (*n* = 14)		iOS (*n* = 15)		Total (*n* = 29)	
			*n*	%	*n*	%	*n*	%
BCT	Tracking	Nutrients	9	64.3	6 (2)	40 (13.3)	15 (2)	51.7 (6.9)
		Calories	10	71.4	13 (2)	86.7 (13.3)	23 (2)	79.3 (6.9)
		Steps	2 (2)	14.3 (14.3)	10	66.7	12 (2)	41.4 (6.9)
		Activity	7 (1)	50 (7.1)	8 (2)	53.3 (13.3)	15 (3)	51.7 (10.3)
		Food	12	85.7	10 (1)	66.7 (6.7)	22 (1)	75.9 (3.4)
		Water	7(2)	50 (14.3)	8 (1)	53.3 (67)	15 (3)	51.7 (10.3)
		Weight	12	85.7	12 (1)	80 (6.7)	24 (1)	82.8 (3.4)
	Motivation	Rewards	0	0	3 (3)	20 (20)	3 (3)	10.3 (10.3)
		Monitoring	0 (1)	0 (7.1)	14	93.3	14 (1)	48.3 (3.4)
		Notifications	0 (2)	0 (14.3)	2 (6)	13.3 (40)	2 (8)	6.9 (27.6)
		Community	5	35.7	8 (1)	53.3 (6.7)	13 (1)	44.8 (3.4)
		Performance management	3	21.4	2 (5)	13.3 (33.3)	5 (5)	17.2 (17.2)
Information		Health	3 (1)	21.4 (7.1)	4 (6)	26.7 (40.0)	7 (7)	24.1 (24.1)
		Nutrition	9	64.3	4 (5)	26.7 (33.3)	13 (5)	44.8 (17.2)
		Calories	10	71.4	12 (1)	80 (6.7)	22 (1)	75.9 (3.4)
		Menu	0 (3)	0 (21.4)	2 (4)	13.3 (26.7)	2 (7)	6.9 (24.1)
Function		Food image recognition	4	28.6	2	13.3	6	20.7
		Meal reminders	10	71.4	6	40	16	55.2
		Sync with other apps	2 (2)	14.3 (14.3)	10	66.7	12 (2)	41.4 (6.9)
		Nutrition report	10 (3)	71.4 (21.4)	10 (2)	66.7 (13.3)	20 (5)	69 (17.2)
		Wearable	0	0	5	33.3	5	17.2

Numbers in parentheses indicate data for apps with paid content. BCT, behavior change technique.

**Table 2:** MARS scores of individual apps: best and worst apps.

	App Name	Platform	Objective Quality				Subjective Quality	Overall Score
			Engagement	Functionality	Aesthetics	Information		
Best 5								
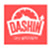	God of Weight Loss—a Diary for Diet and Exercise	Android	4.3	4	4	4	4	4.1
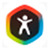	Automatic Pedometer	iOS	4	4.1	4.3	4	4	4.1
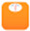	Lost It!	iOS	4	4	3.9	3.9	4.3	4
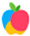	YAZIO Coach	Android	4.2	3.7	4.1	4	3.2	3.9
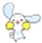	Friends on a Diet	iOS	4.5	3.6	3.5	3.8	3.8	3.8
Worst 5								
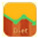	Easy Weight Manager	Android	2.1	1.8	2.3	1.9	1.3	1.9
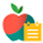	iEatWell: Food Diary	Android	2.3	1.3	2.7	2.2	1.3	1.9
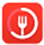	Intermittent Fasting	iOS	1.4	2	1.7	2	1.7	1.8
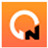	Fitness Diary	iOS	1.4	2	1.7	1.7	1.1	1.6
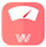	Freeze Weight	Android	1.3	1.4	1.2	1.8	1.2	1.4

**Table 3:** Regression analyses: association between app content (tracking, motivation, information, and function) and MARS scores.

Model	*B*	SE	*β*	*t*	*p*
Tracking	0.031	0.079	0.072	0.390	0.700
Motivation	0.131	0.063	0.261	2.090	0.047
Information	0.311	0.102	0.573	3.035	0.006
Function	0.053	0.087	0.090	0.608	0.549
Intercept	1.732	0.238		7.281	0.000

F = 15.058, R^2^ = 0.715, adj. R^2^ = 0.668.

## Data Availability

The data presented in this study are available on request from the corresponding author.
